# Expression and Function of Host Defense Peptides at Inflammation Sites

**DOI:** 10.3390/ijms21010104

**Published:** 2019-12-22

**Authors:** Suhanya V. Prasad, Krzysztof Fiedoruk, Tamara Daniluk, Ewelina Piktel, Robert Bucki

**Affiliations:** Department of Medical Microbiology and Nanobiomedical Engineering, Medical University of Bialystok, Mickiewicza 2c, Bialystok 15-222, Poland; suhanyavp@gmail.com (S.V.P.); krzysztof.fiedoruk@umb.edu.pl (K.F.); tamara.daniluk@umb.edu.pl (T.D.); ewelina.piktel@wp.pl (E.P.)

**Keywords:** host defense peptides, human antimicrobial peptides, defensins, cathelicidins, inflammation, anti-inflammatory, pro-inflammatory

## Abstract

There is a growing interest in the complex role of host defense peptides (HDPs) in the pathophysiology of several immune-mediated inflammatory diseases. The physicochemical properties and selective interaction of HDPs with various receptors define their immunomodulatory effects. However, it is quite challenging to understand their function because some HDPs play opposing pro-inflammatory and anti-inflammatory roles, depending on their expression level within the site of inflammation. While it is known that HDPs maintain constitutive host protection against invading microorganisms, the inducible nature of HDPs in various cells and tissues is an important aspect of the molecular events of inflammation. This review outlines the biological functions and emerging roles of HDPs in different inflammatory conditions. We further discuss the current data on the clinical relevance of impaired HDPs expression in inflammation and selected diseases.

## 1. Introduction

The human body is in a constant state of conflict with the unseen microbial world that threatens to disrupt the host cell function and colonize the body surfaces. The immune system has an arsenal of destructive mechanisms to neutralize the toxic effect of the microbial pathogens. It functions through two layers of defense systems: The innate system and the more intricate adaptive immune system, which closely communicate with each other [1]. Each of those systems form a complex network of immune cells, signaling molecules, and regulatory pathways. Inflammation is a reaction of the host immune system that acts to eliminate the source of inflammatory stimulus, ranging from pathogens to burn injuries [2]. Although microbial infections largely initiate the events of inflammation, we must note that inflammation is also a hallmark feature of various autoimmune, cancer, and systemic diseases [3]. Inflammation is a highly coordinated biochemical sequence of events that commences with the rapid migration of leukocytes to the site of infection, followed by adequate blood supply that transports different inflammatory mediators that control the course of the immune response [4]. However, while the initial events of inflammation are constructive and beneficial to the host, incompetent inflammatory resolution mechanisms, along with inefficient elimination of foreign bodies or pathogens and cellular debris, prompt the onset of chronic inflammation.

Antimicrobial peptides (AMPs) such as defensins and cathelicidins represent a vital part of the human immune system due to their broad spectrum activity against pathogenic bacteria, fungi, protists, and enveloped viruses [5]. Furthermore, in recent years, a growing number of studies have recognized these peptides as potent immune modulators, implicated in multiple pro- and anti-inflammatory responses through (1) neutralization of bacterial toxins, (2) chemoattraction and activation of immune cells, (3) initiation of adaptive immunity, (4) neovascularization and wound healing, as well as (5) anti- or pro-tumor activity [6]. Actually, AMPs interact with innate and adaptive immune receptors, such as pattern recognition (PRRs) or chemokine receptors (CCRs), as well as inflammasomes and their complement systems, creating a link between innate and adaptive immunity [7,8,9]. In addition, AMPs may regulate fundamental cellular processes, such as differentiation, proliferation, and programmed cell death, e.g., by stimulating growth factor receptors or as complexes with the host nucleic acids [10,11], hence they resemble cytokines and growth factors. Overall, these activities, by controlling inflammation and/or accelerating repair process of the infected site, appear to support the direct microbicidal function of AMPs in resolving an infection. Therefore, the term host defense peptides (HDPs) was coined to encompass their pleiotropic nature, and association with both infectious as well as non-infectious inflammatory responses [12], although these two terms are used interchangeably. In the latter context, HDPs fit into the definition of “alarmins” or “danger signals”, i.e., various endogenous molecules collectively known as DAMPs (damage associated molecular patterns), which are released from damaged or dying cells and initiate a diverse range of physiological and pathophysiological functions [13,14,15]. 

**Figure 1:**
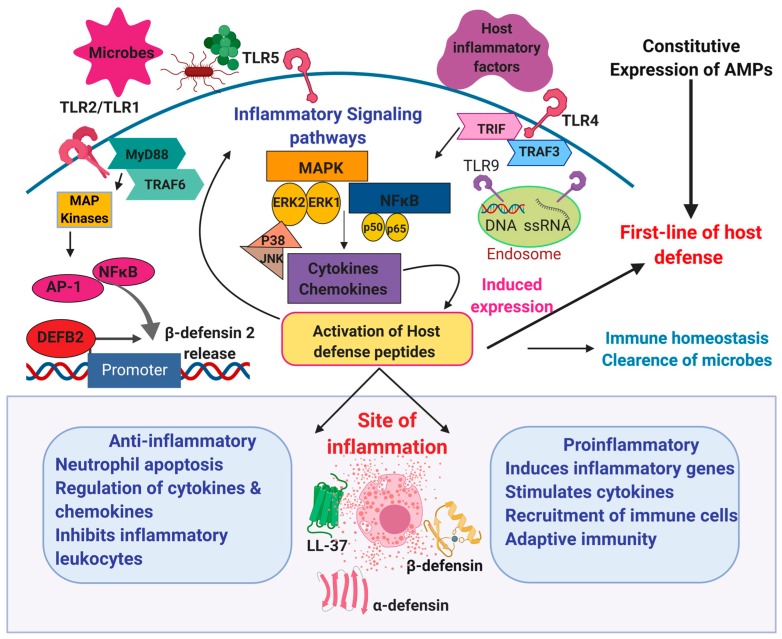
Illustration of the distinct role of host defense peptides at the sites of inflammation. Specific Toll-like receptors TLR-2, TLR-4, TLR-5, and TLR-6 are expressed on the plasma membrane of immune cells, non-immune cells, and intracellular compartments. TLR-7 and TLR-9 within endosomes participate in the host recognition of microbial cellular components and bind to host internal factors. The receptors, along with their adaptor proteins MyD88 and TRIF, can initiate the inflammatory signaling pathways. The host defense peptides promote innate immunity against various pathogens and maintain the immune system homeostasis. These peptides can also be induced in addition to their constitutive expression by the transcriptional modulatory factors NF-κB, AP-1, and intracellular release of cytokines and chemokines. They actively participate in coordinating the host immune signaling mechanisms during inflammation. Furthermore, HDPs can display both pro- and anti-inflammatory properties that may protect against the responses of inflammatory diseases. Abbreviations: AP-1, activator protein; NF-kB, nuclear factor kappa-light-chain enhancer of activated B cells; MAPK, mitogen-activated protein kinase; ERK1,2, extracellular signal-related kinases.

At present, HDPs are perceived as multifunctional agents that coordinate diverse immune surveillance functions necessary to maintain homeostasis (Figure 1) [16]. However, if their production is out of the physiological range, they may contribute to an undesirable inflammation in response to local (e.g., periodontal, respiratory, intestinal, and skin) and systemic (e.g., sepsis) infections. They might also function as pathophysiological events of inflammatory diseases, cancers, and even psychiatric disorders (Table 1) [17,18,19,20,21,22,23]. In addition, HDPs have been indicated as potential biomarkers in numerous infectious and non-infectious diseases [24].

That being said, expanding our knowledge regarding the molecular mechanisms behind expression, processing, and mutual interactions of HDPs with other immune system components is crucial to better understand inflammation processes, and to develop new methods of anti-inflammatory treatment. Certainly, it is a challenging and long-term task, since a single antimicrobial peptide, e.g., human cathelicidin LL-37, may interact with dozens of proteins/receptors and subsequently engage hundreds of secondary effector proteins, as well as modify expression of >900 genes [6]. Considering this, the purpose of this review is to evaluate and summarize recent discoveries considering the functional expression and protective attributes of HDPs/AMPs in the acute inflammatory phase and the detrimental effects of their recruitment in chronic inflammation. Both in vitro and in vivo studies connecting the underlying mechanisms governing the immunoregulatory role of these peptides in the inflammatory microenvironment will be discussed.

## 2. Overview of Human Antimicrobial Peptides

**Figure 2:**
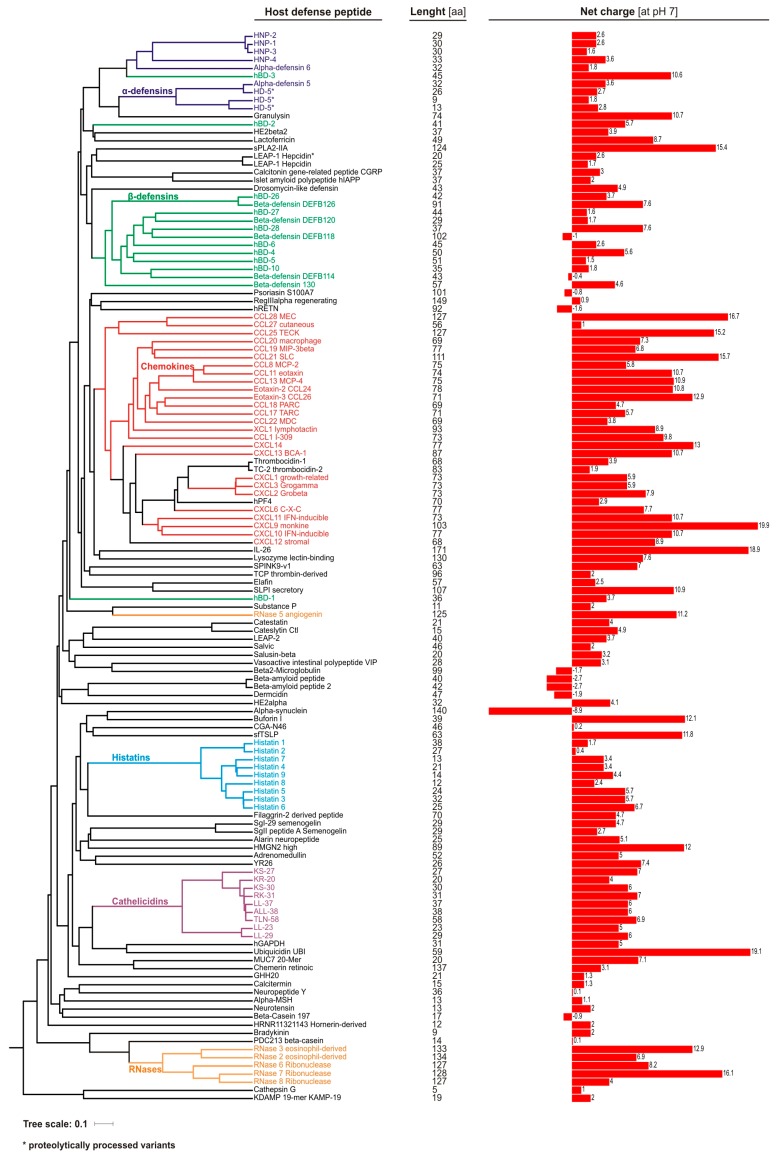
Comparison of human host defense peptides (*n* = 133) curated in antimicrobial peptide database (http://aps.unmc.edu/AP; accessed in September 2019). The dendrogram was built based on amino acid sequence alignment using MAFFT aligner (https://mafft.cbrc.jp) [65], visualized and annotated with Archaeopteryx [66] and iTOL [67], respectively. Net charge values of the peptides (at pH = 7.0) were estimated using Protein Calculator https://pepcalc.com/protein-calculator.php [68].

Antimicrobial peptides are widely distributed in all living organisms, representing ancient and primary defense molecules, e.g., innate immune mechanisms conferred by the antimicrobial peptides in insects usually devoid of adaptive responses [25]. Discovery of defensins in rabbit leucocytes, lactoferrin in cow milk, and lysozyme in human saliva are among the first reports of animal-originated antimicrobial molecules, which paved the way for further identification and understanding of the physiological function of other antimicrobial peptides and proteins [26]. At present, 2272 peptides derived from animals, including ~130 of human origin (Figure 2), are recorded in the antimicrobial peptide database (http://aps.unmc.edu/AP), a comprehensive source of naturally existing families of antimicrobial peptides from all form of kingdoms of life [25].

Antimicrobial peptides and proteins contain a short chain of about 12–100 amino acids (Figure 2), and are classified according to their conformational structure (α, β, αβ, and non-αβ), amino acid motifs, and expression pattern [25,27]. For example, the major human AMPs, cathelicidin LL-37 and defensins, are characterized by α-helical and β-sheet structure, respectively. Furthermore, the latter are divided into α- and β-defensins based on the configuration of the disulfide bonds between six cysteine residues. AMPs are characterized by positive charge and substantial proportion (typically 50%) of hydrophobic residues, thus they are also known as cationic antimicrobial peptides (CAPs). However, at present, some negatively charged peptides are also classified as AMPs, e.g., human β-defensin DEFB118, psoriasin, or α-synuclein (Figure 2). Nevertheless, this amphiphilic–cationic organization allows them to selectively associate, and in turn disrupt, highly negatively charged microbial membranes. Hence, it explains their broad spectrum of activity, encompassing all cellular pathogens and enveloped viruses. Additionally, the cationic nature of AMPs may possibly facilitate, via electrostatic forces, their interactions with diverse host receptors, which are behind the immunomodulatory potential of these peptides [28].

Certain AMPs, e.g., cathelicidins, are produced as inactive pro-peptides and must be proteolytically processed for activity. It is noteworthy that this may generate multiple length variants characterized by diverse antimicrobial or immunomodulatory properties. Therefore, the presence of the appropriate proteases and their level is an important factor in regulating the function of the AMPs. Another important activity-related issue is that microbicidal action of AMPs is considerably suppressed by the physiological conditions present in some compartments of the body, including high salt, carbonate, lipoprotein, and polysaccharide concentrations [29,30,31,32,33].

The sensitivity to environmental factors of these peptides was well illustrated by the inability to reproduce the protective role of insect-derived AMPs, such as drosocin, in a mouse model [52]. Briefly, the authors explained this difference by an unusually high degradation rate of such peptides in mammalian sera (human and mouse) in comparison to insect hemolymph. In contrast, physiological conditions have no impact on the immunomodulatory properties of AMPs, such as chemoattraction or activation of immune cells. In addition, the antimicrobial activity of AMPs estimated in vitro, i.e., MIC (minimal inhibitory concentration) values, is usually observed at micromolar concentrations which are significantly higher than the physiological concentrations of these peptides. For instance, the concentration of LL-37 or β-defensins is less than 2 μg/mL at mucosal sites, and the MIC of LL-37 in vitro against *Escherichia coli* is more than 32 μg/mL [10], whereas modulation of immune responses by AMPs occurs at nanomolar levels [53]. Therefore, it is possible that the other biological functions of AMPs, e.g., as alarmins, may play more prominent roles than their direct microbicidal effects in combating invading pathogens in vivo [6,10,53]. Indeed, several synthetic AMP derivatives, known as innate defense regulator (IDR) peptides, are characterized by potent immunomodulatory activities [54].

In fact, certain human AMPs such as the histone protein H2A (known as buforin I) or ribosomal protein S30 (known as ubiquicidin) were initially known from non-antimicrobial functions, before their antimicrobial potential was recognized. In addition, around 20% of human AMPs (Figure 2) are chemokines, which as cationic and amphipathic molecules are characterized by antimicrobial activity [55]. In addition, the specific chemokine receptor CCR6, expressed by dendritic cells and T cells, is utilized also by human β-defensin-2 peptide [56], supporting the hypothesis that AMPs create a bridge between innate and adaptive immune system.

AMPs protect all human body sites that are continually exposed to microbes, like the skin and mucous membranes, since they are produced by multiple immune and epithelial cells (Table 1). Their expression may be constitutive and some cells (e.g., neutrophils) store a high number of AMPs, or the expression is induced by various microbial or the host stimuli. As a consequence, each tissue has its own profile of different AMPs that may vary significantly depending on the actual host condition. It is tempting to name it as a “peptidiome” using an analogy to microbiome bacteria within a given body habitat. Therefore, (1) a synergism of AMPs activity, supported by their (2) accumulation, e.g., in neutrophil extracellular traps (NETs) (see below), as well as (3) enhanced expression, may explain the insufficient microbicidal concentration issue observed at the basal physiological background. On the other hand, this effect may just be a derivative of inadequate in vitro MIC testing methods. For example, Dorschner et al. [57] showed that cultivation of bacteria, like *Staphylococcus aureus* and *Escherichia coli*, in a medium mimicking the mammalian ionic environment, i.e., carbonate-containing solutions, causes changes in their cell wall thickness and an altered gene expression pattern, that in turn increased susceptibility to AMPs. Furthermore, it is possible that in the skin or inside phagocytic cells, i.e., body niches where the level of the AMPs-inhibiting factors is minor, this antagonism is not significant.

### 2.1. Human Defensin and Cathelicidin (LL-37) Peptides

Defensins are cysteine-rich peptides classified based on configuration of the disulfide bonds between six cysteine residues into α-, β-, and θ-defensins; however, in humans, the latter exist only as pseudogenes [58]. From the evolutionary perspective, β-defensins are the common ancestor of all vertebrate defensins, and α-defensins are mammalian-specific genes co-located with β-defensin ones on adjacent loci on human chromosome 8p22–p23 [59,60]. Human α-defensins are produced mainly by neutrophils; hence, they are known as human neutrophil peptides 1–4 (HNP-1, HNP-2, HNP-3, and HNP-4), as well as by Paneth cells of the small intestine (HD5 and HD6) (Table 1) [61,62]. Interestingly, the four HNPs are encoded by three genes, since HNP-2 is a truncated variant of HNP-1 or HNP-3 peptides, lacking the first alanine or aspartic acid residue, respectively [63]. HNP1–4 are constitutively expressed and stored in azurophil granules, where they constitute more than 30% of the protein content; however, HNP-4 is the least abundant [61,64].

In contrast, at least 17 β-defensins (hBDs) have been described, yet hBD1–hBD4 are the best studied [69]. They are produced by various epithelial and mucosal cells, thus protecting body sites directly exposed to microbes, such as respiratory, intestinal, and genitourinary tracts, as well as skin (Table 1), where their expression may be constitutive or inducible. For example, expression of the *hBD-1* gene is essentially constitutive, whereas expression of the *hBD2*-*4* genes is infection-related or triggered by host-derived stimuli [70]. Remarkably, the microbicidal effect of hBD-3 peptide is not weakened in the presence of the physiological salt concentration found in mucus, which enables it to have a substantially strong anti-HIV effect [71]. In addition, ß-defensin genes *(DEFB4, DEFB103,* and *DEFB104*) have a high degree of copy-number variation (CNV), ranging from 2 to 12 copies per diploid genome [72], which affects their expression level.

Cathelicidins were named based on a conserved cathelin-like domain connected with a C-terminal antimicrobial domain, and are produced mainly by leucocytes and epithelial cells [73,74]. In the human genome, only one cathelicidin gene (*CAMP*) is present. Nevertheless, as the result of proteolytic cleavage by various proteases of its product, i.e., hCAP-18 (human cationic antimicrobial protein 18 kDa), several cathelicidin peptide variants are generated (Figure 2). In detail, in the first step, hCAP-18 is processed by protease 3 to the full-length active peptide LL-37 (leucine–leucine 37 aa), which in turn is cleaved into shorter variants by tissue-specific proteases. In the skin, serine proteases from the kallikrein family, SCTE (stratum corneum tryptic enzyme; kalikrein 5) and SCCE (stratum corneum chymotryptic enzyme; kalikrein 7), generate peptides KS30, KS22, LL29, and RK31 and KR20, respectively [75]. In fact, in the skin, LL-37 accounts for less than 20% of all cathelicidin variants. Interestingly, KS30, KS22, and LL29 are characterized by stronger antimicrobial activity, but lack of chemotactic properties. On the other hand, RK31 and KR20 peptides possess weak antibacterial but strong antifungal activity. Recently, also the TLN-58 variant, possibly generated by neutrophil elastase (ELA2), has been found in the skin palmoplantar pustulosis (PPP) vesicles [76]. Furthermore, since hCAP-18 is present in semen, a longer, by an additional alanine residue, peptide ALL-38 is produced as the result of action of prostate-derived protease, gastricsin, under acid vaginal pH conditions.

### 2.2. Other Host Antimicrobial Peptides

Besides the classical antimicrobial peptides, there is an array of small proteins regulating immunomodulatory and antimicrobial functions against a broad range of pathogens. For instance, histatin, lysozyme, hepcidin, thrombocidin-1, neuropeptide α-MSH, RNase 7, RNase 5, and dermcidin are inherently expressed in specific tissues and cells (Table 1). Briefly, histatins 1, 3, and 5 belong to a family of salivary peptides that help to maintain the human oral mucosa, along with the β-defensins. An elevated expression of histatin 5 is detected in the saliva of children with a high level of dental cavities harboring specific bacterial species, such as *Streptococcus mutans*, *S. sanguinis*, *S. mitis,* as well as *Lactobacillus rhamnosus* in the oral environment [77,78]. In contrast, RNAse 7 is abundantly found within specialized uroepithelial cells in bladder lining, ureters, and kidneys, protecting the urinary system from invading microbes. This peptide exhibits a significant role in maintaining a bacteria-free bladder, as it inhibits the microbial activity of various drug-resistant microbes, including *Klebsiella pneumoniae,* *Pseudomonas aeruginosa,* and vancomycin-resistant *Enterococcus faecium* [79]. Another important antimicrobial peptide synthesized in the liver is hepcidin. While its primary function involves maintenance of iron absorption and transport, hepcidin also exhibits strong antimicrobial activity. During inflammatory conditions, hepcidin mRNA expression is highly stimulated by the cytokines IL-6, IL-1α and IL-1β, which modulates host response [80]. 

## 3. Role of Host Defense Peptides in Inflammation

Over the years, our view on antimicrobial peptides (AMPs) has evolved from just endogenous antibiotics into multifunctional agents (HDPs), which execute their antimicrobial tasks at the same time as participating in a pro-inflammatory response and, if required, mediating its suppression. Currently, HDPs are perceived as factors contributing either to efficient clearance of infections or resolution of the infected sites. To illustrate, these peptides not only attract immune cells, e.g., neutrophils, but also by blocking apoptosis prolong their lifespan, and in turn phagocytic functions [81]. On the other hand, HDPs may function as a “molecular brake” on macrophage-driven inflammation to maximize eradication of pathogens with minimal adverse effects on surrounding tissues [82].

Furthermore, HDPs are essential for proper host–microbiota interactions. In this context, HDPs serve as a buffer, maintaining immune homeostasis via neutralization of pro-inflammatory MAMPs, e.g., lipopolysaccharides (LPS) and lipoteichoic acid (LTA), constantly released by microbiota, as well as a factor shaping its composition, hence protecting from dysbiosis [83]. For example, LL-37 inhibits the expression of specific pro-inflammatory genes up-regulated by NF-κB in the presence of LPS, unlike to LPS-induced genes which antagonize inflammation and certain chemokine genes classically considered pro-inflammatory [84]. On the other hand, the microbiota are a key factor in stimulating production of HDPs, as supported in a classical experiment by Mangoni et al. [85], showing that the presence of HDPs in frog skin (*Rana esculenta*) is microbiota-dependent, and frogs living in a sterile, i.e., the microbiota-free, environment do not synthesize antimicrobial peptides.

**Figure 3:**
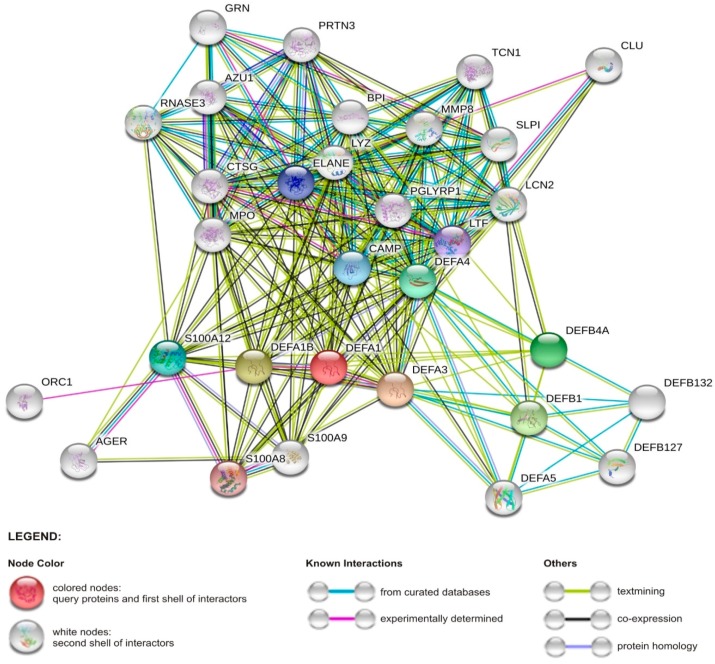
Network of interactions between α-defensin 1 gene (*DEFA-1*) and other human proteins (the network was obtained from STRING v11 database) [87]. Represented proteins are central to antimicrobial and immunomodulatory activities. Abbreviations: AGER, advanced glycosylation end-product-specific receptor; BPI, bactericidal permeability increasing protein; CAMP, cathelicidin antimicrobial peptide; CLU, clusterin; CTSG, cathepsin; DEFA3, defensin alpha 3; DEFA4, defensin alpha 4; DEFA5, defensin alpha 5; DEFA1B, defensin alpha 1B; DEFB4A, defensin beta 4A; DEFB1, defensin beta 1; DEFB132, defensin beta 132; DEFB127, defensin beta 127; ELANE, neutrophil elastase; GRN, granulin precursor; LCN2, lipocalin 2; LTF, lactotransferrin; LYZ, lysozyme; MPO, myeloperoxidase; MMP8, matrix metallopeptidase 8; ORC1, origin recognition complex subunit 1; PGLYRP1, peptidoglycan recognition protein 1; PRTN3, proteinase 3; RNASE3, ribonuclease A family member 3; SLP I, secretory leukocyte peptidase inhibitor; S100A8, S100 calcium binding protein A8; S100A9, S100 calcium binding protein A9; S100A12, S100 calcium binding protein A12; TCN1, transcobalamin 1.

Importantly, HDPs inhibit not only pro-inflammatory action of exogenous PAMPs, but also endogenous ones, like DAMPs (also known as “alarmins” or “danger signals”), which are expressed in stressed or dying cells and convey alarm signals to the immune system, including those responsible for autoimmune disorders [13]. For instance, in the skin cathelicidin peptides block release of cytokines induced by the alarmin hyaluronan, thus their low expression may be a risk factor of the development of atopic dermatitis [86]. Initially, the term DAMPs involves factors from various cell/tissue compartments, such as extracellular matrix (hyaluronan, heparan sulfate, eDNA), cytoplasm (heat shock proteins: HSP60, HSP70, HSP90, HSP27; calcium-binding proteins: S100A8, S100A9, S100A12; β-Galactoside binding lectins: Galectin-1, Galectin-3; and uric acid), mitochondria (mitochondrial DNA, ATP, N-formylated peptides), and other subcellular organelles (HMGB1, IL-33, IL-1α, Calreticulin). However, currently, several HDPs, e.g., cathelicidins and α- and β-defensins, are also classified as alarmins [14], which in fact are frequently co-expressed with DAMPs (Figure 3).

Indeed, a link between HDPs and multiple autoinflammatory diseases such as skin disease (atopic dermatitis, psoriasis, rosacea) or microbiota-related ones, e.g., IBD (Crohn’s disease, colitis ulcerosa), acne vulgaris, and periodontitis, has been established by several studies (see below). An enhancement of Th17 response by HDPs may serve as an example. Briefly, HDPs efficiently attract Th17 (T helper 17 cells), which in turn secrete pro-inflammatory cytokines, IL-17A, IL-17F, IL-21, and IL-22, responsible for mounting mucosal defense against pathogenic microbes in the respiratory or intestinal tract. For instance, IL-17A and IL-22 work synergistically to induce certain β-defensins hBD-1, hBD-3, and hBD-4 in both human and primary mouse gastric epithelial cells (GEC) and gastroids co-cultured with *Helicobacter pylori* [88]. On the other hand, an elevated level of Th17 cells has been connected with various autoimmune diseases, such as systemic lupus erythematosus, rheumatoid arthritis, or psoriasis [89].

Also, genetically-mediated deficiency/excess of HDPs, gene sequence polymorphisms, as well as disturbed expression may be a risk factor in inflammatory diseases. For instance, Hollox et al. [90] showed a significant association between higher genomic copy numbers for β-defensin genes, ranging from 2 to 7 copies, and the relative risk of developing psoriasis. Likewise, a lower the *hBD-2* gene copy number in the β-defensin locus predisposes to colonic Crohn’s disease [91]. Recently, experimental evidence has highlighted the genetic association between the clinical phenotype of sepsis and *DEFA-1*/*DEFA-3* copy number. Transgenic mice models were engineered to produce a high gene copy number of *DEFA-1*/*DEFA-3*, which manipulated the outcome of sepsis progression [92]. The consequential effect was compared to the low gene copy number wild-type mice models, in that the former showed chronic inflammation, endothelial cell damage, vascular leakage, severe organ injury, and mortality. Thus, treatment of patients with sepsis can be challenging due to the underlying individual genetic associations. However, further research is needed to obtain conclusive data. In addition, single nucleotide polymorphisms (SNPs) of the *hBD-1* gene was connected with the pathogenesis of inflammatory bowel diseases and chronic gastritis [93], as well as oral diseases [94].

In general, expression of HDPs is enhanced during infection or inflammation through transcription factors initialized by pro-inflammatory cytokines or signaling pathways associated with activation of PRRs, e.g., Toll-like receptors (TLRs). For instance, promoter regions of α- and β-defensin genes contain binding sites for major cellular transcription factors, notably nuclear factor κB (NF-κB) and activator protein 1 (AP-1) (Figure 1). It should be noted that NF-κB also plays a crucial role in the pathogenesis of Crohn’s disease, along with many other pro-inflammatory molecules that modulate the hBD-2 expression [95], as well as in triggering its production (and IL-6) in severe sepsis [96]. Moreover, the expression of genes encoding LL-37 (and hBD-2) is modulated by vitamin D3 via binding with specific DNA sequences in their promoters, the so-called vitamin D response elements (VDRE) [97]. Additionally, a recent in silico analysis identified a wide range of transcription factors which possibly bind and modulate the gene transcription of many antimicrobial peptides and proteins, such as LL-37, RNAse1, CCL18, CXCL14, and HTN1 [98].

In line with this, it has been established that DNA methylation of the CpG sites in the 5′ flanking region of the *hBD-1* gene contributes to its deficiency in patients with atopic dermatitis [99]. Furthermore, point mutations in the promoter region of *hBD-1* explain a cancer-specific loss of this peptide in 90% and 82% of renal cell carcinomas and prostate [100]. Thus, *hBD-1* was suggested as a potential tumor suppressor gene for urological cancers. Also, in oral squamous cell carcinoma (OSCC), hBD-1 appears to have anti-tumor properties, while *hBD-2* and *hBD-3* might be proto-oncogenes [101].

Nonetheless, the relation between HDPs and inflammation is not always straightforward, and either their deficiency or overproduction, as well as a balance between pro- or anti-inflammatory effects, may contribute to the pathological inflammatory response. For instance, in atopic dermatitis (AD), despite severe skin inflammation, the expression level of major skin HDPs, dermcidin, LL-37, hBD-2, and hBD-3, is not increased, hence patients with AD are more prone to skin infections and have altered skin colonization patterns. By contrast, in psoriasis, expression of LL-37, hBD-2, and hBD-3 is elevated, hence skin infection is rare. Nevertheless, LL-37 and hBDs are considered as a major driving force of inflammation in psoriasis by mechanisms involving increased production of IFN-α and activation of pDCs, respectively [102]. In addition, these peptides stimulating degranulation of mast cells and increasing production of the pruritogenic cytokine IL-31 may escalate itching (pruritus) manifestation [103]. However, it has been recently observed that LL-37 may also act as an anti-inflammatory agent by blocking the release of inflammatory cytokine IL-1β, depending on its concentration. Interestingly, this observation possibly explains the mechanism underlying the paradoxical effectiveness of vitamin D3, i.e., inducer of LL-37 expression, in treatment of psoriasis [11]. Similarly, hBD-3 may inhibit inflammation by inducing expression of anti-inflammatory cytokine IL-37 in keratinocytes [104]. An elevated level of cathelicidin is also observed in other inflammatory skin conditions, namely rosacea and palmoplantar pustulosis, but instead of the native form of LL-37, its proteolytically cleaved variants drive the inflammation [76,105].

**Figure 4:**
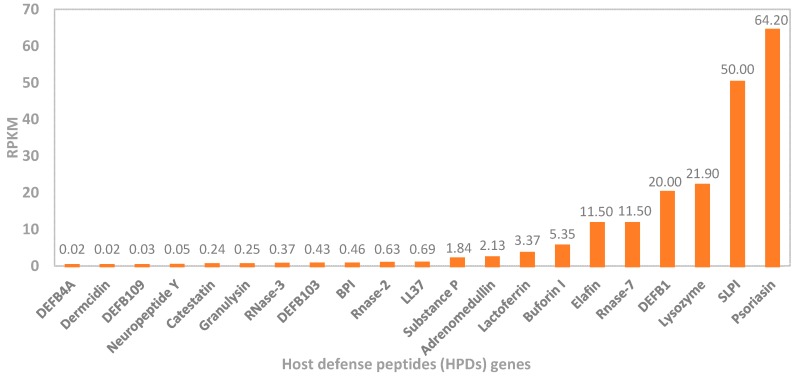
Relative expression of selected host defense peptide (HDP) genes in the human skin, based on RNA-seq analysis of tissue samples from 95 human individuals performed by Fagerberg et al. [107]. The data were obtained from the GenBank Bio Project: PRJEB4337 (RPKM, reads per kilobase per million reads).

Considering the above, the final contribution of HDPs to inflammatory processes is a derivative of multiple variables related to their expression, processing, concentration, and combination, as well as reciprocal interactions with the remaining components of the host immune system. For instance, pro- and anti-inflammatory effects of cathelicidin LL-37 are concentration-dependent, i.e., the former is visible at >20 µg/mL, whereas the latter at 1–5 µg/mL. Similarly, at the concentration range 1–100 ng/mL, β-defensins can act as chemokines only, since other immunomodulatory functions are not visible [10]. Furthermore, there are more than 20 antimicrobial peptides in the skin characterized by different expression levels (Figure 4) [106]. Therefore, the relationship between HDPs and inflammation appears to be strongly context-dependent (e.g., inflammation site, type of cells, or stimuli), and as such, it should be analyzed on a multidimensional level, rather than as a single action of individual peptides. Otherwise, valid conclusions regarding the ultimate role of HDPs in inflammation may be difficult to draw. In the next paragraph, we discuss mechanisms behind anti- and pro-inflammatory actions of HDPs.

## 4. Molecular Mechanisms of Anti- and Pro-Inflammatory Action of HDPs

The immunomodulatory potential of HDPs is strictly connected with their ability to recruit and activate immune and non-immune cells, as well as a direct or indirect impact on their fate, including maturation, differentiation, degranulation, or apoptosis [10]. This is mediated through interaction with a wide range of membrane-bound and intracellular receptors, followed by stimulation of their downstream signaling pathways. So far, HDPs have been recognized to interact with the following receptors: (1) Pattern recognition receptors (PRRs), (2) purinergic G-protein coupled receptors (formyl peptide receptor like-1), (3) P2X7 receptor, (4) MRGPRX2, (5) chemokine receptors (commonly known CCR2, CCR6), (6) epidermal growth factor receptors (tyrosine kinases), (7) integrin family receptors (macrophage-1 antigen), nucleotide oligomerization domain (NOD) proteins, and NODlike receptors (NLRs), and their number is still growing [108,109]. Hence, HDPs modulate immune responses using the same receptors as MAMPs/PAMPs and DAMPs [110,111]. To illustrate, β-defensins attract cells by interaction mainly with CCR2 and CCR6 receptors, and exert their “alarmin” activity, e.g., induction of cytokine production, via TLRs, EGFR, GPCR, and MrgX2 ones; however, both activities may overlap in one receptor.

It should be noted that TLR receptors may be the root cause of certain HDP-associated diseases. For instance, in individuals with rosacea, a higher expression of TLR-2 sensitizes the facial skin to microbes or environmental stimuli. Under these conditions, enhanced expression of kallikrein-5 proteinase is observed in keratinocytes, and ultimately affects production of cathelicidin peptides, which drives inflammation and abnormal growth of blood vessels [105]. Moreover, the tumor-suppressing effect of hBD-1 is associated with its ability to modulate epidermal growth factor and human epidermal growth factor receptor 2 (EGFR/HER2)-associated signaling pathways [112]. Finally, hBD-3, through deactivation of TLR-4 and TLR-2, may reduce the adverse immune reaction initialized by NF-κB in response to LPS [113].

Furthermore, β-defensins and cathelicidins, in the same manner as PAMPs (e.g., LPS) and DAMPs (e.g., heat shock antigens Hsp60 and Hsp70), are ligands of Toll-like receptor 4 (TLR-4). However, the resulting outcome of the receptor’s stimulation may be different for these molecules. For instance, unlike LPS, hBD-3 does not induce production of IL-10, which is an important anti-inflammatory cytokine, e.g., via suppressing function of antigen-presenting cells (APCs), suggesting that hBD-3 can shift the immune response toward pro-inflammatory direction [114]. Similarly, hBD-2 via TLR-4 leads to maturation of dendritic cells (DCs), which consequently exhibit Th1-polarized responses, such as the production of pro-inflammatory cytokines IL-12, IL-1α, IL-1β, and IL-6, which may possibly counter suppressive action of microbial factors by generating more robust host inflammatory and Th1 responses [115]. In contrast, cathelicidin is considered as an inhibitor of TLR-4, and thus can antagonize with other TLR-4 ligands released during skin injury, e.g., hyaluronan [10].

Therefore, HPDs joining properties of MAMPs/PAMPs and DAMPs may operate as central nodes in a network that coordinates immune response to infections as well as non-infectious insults. For instance, it has been shown that synergistic action of MAMPs/PAMPs and DAMPs is necessary for synthesis and subsequent secretion of pro-inflammatory cytokine IL-1β [13]. It is important to note that a lack of IL-1β results in high susceptibility to infections, but its overproduction causes uncontrolled inflammation and tissue damage via T cell-mediated autoinflammatory response [116]. Indeed, overproduction of IL-1β was observed in patients with inflammatory bowel disease, and has been connected with deficiency of α-defensins that serve as regulators of IL-1β maturation [117]. As aforementioned, also in psoriasis, LL-37 may act as an inhibitor of the IL-1β release in keratinocytes by blocking activation of the cytosolic DNA-sensing signaling AIM2, i.e., cytosolic receptor for dsDNA. Hence, cytoplasmic DNA appears to contribute to the pathogenesis of psoriasis via activation of IL-1β in keratinocytes by AIM2-mediated inflammasomes [11].

In this context, it is relevant to mention the relationship of HDPs and self-nucleic acids, and its impact on inflammation. Under normal homeostatic conditions, the host-derived nucleic acids released from damaged and dying cells do not mediate inflammatory responses because of the systematic regulation and physiological location of nucleic acid sensing TLR7/9. However, several studies have shown that HDPs may disturb immune tolerance to self-nucleic acids, and in turn significantly enhance cell responses—in particular, plasmacytoid dendritic cells (pDCs) [118]. In fact, in the skin, this mechanism has been identified as an important initiator of psoriasis development, where LL-37 and defensins are able to condense self-DNA into particles, which are internalized by pDCs, inducing robust IFN-α response via activation of the TLR-9 signaling pathway [119,120]. This enhances production of large amounts of type I IFN, leading to the functional activation of myeloid dendritic cells (mDCs), monocytes, NK cells, keratinocytes, as well as Th1/Th17 differentiation, which further increase the pro-inflammatory, e.g., IFN-γ, IL-22, and IL-17, cytokine expression [120,121]. In line with this, a novel mechanism of nucleic acid recognition by LL-37 utilizing cell surface RNA scavenger receptors (SRs) has been described [122], which results is enhanced clathrin-dependent endocytosis, facilitating the overproduction of inflammatory cytokines and chemokines. Recently also RNase7 was found to utilize plasmacytoid dendritic cell (pDC) TLR-9 signaling mode of IFN-α activation even more strongly than LL-37, emphasizing its crucial role in autoimmune inflammatory skin diseases [123]. Interestingly, other antimicrobial peptides expressed in the skin, such as psoriasin, elafin, or hBD-1, lack the ability of interacting with the host nucleic acids, which may be related to their lower net charge (Figure 2) [120].

Another interesting consequence of interactions between HDPs and the host nucleic acids is a novel wound healing mechanism, where LL-37 may alter wound repair by modifying the responses to dsRNAs released as a result of skin injury. In detail, LL-37 enhances endosomal uptake of non-coding double stranded RNA in TLR-3-mediated mechanism, that results in activation of several important wound repair growth factors, including fibroblast growth factor (FGF2), and heparin binding EGF-like growth factor (HBEGF) from the dermal keratinocytes and fibroblasts [124]. Inhibition of LL-37/dsRNA relation may contribute to the development of hyperproliferation-based diseases, like psoriasis, whereas its augmentation can lead to increased wound regeneration in pathological conditions of abnormal wound repair (e.g., diabetic ulcers).

Finally, LL-37 actively participates in neutrophil extracellular trap (NET) formation via disruption of the nuclear membrane and promotes their stability [125]. NETs are structures composed of decondensed chromatin and multiple enzymes (elastase, myeloperoxidase, gelatinase, etc.) and proteins, including antimicrobial ones. Thus, NETs act as a mechanical barrier that entraps and subsequently reduces spreading of pathogens and/or their toxic products into the host tissues, where the antimicrobial activity of HPDs is boosted by their accumulation and combination [126]. Moreover, Stephan et al. have shown that complexes of LL-37/DNA formed inside human macrophages may participate in defense against intracellular bacteria, e.g., mycobacteria [127]. Accordingly, a recent study investigated the therapeutic potential of LL-37 in modulating macrophage-mediated excessive inflammatory responses. It was found that LL-37 reduced the severity of tuberculosis by rapidly enhancing the anti-inflammatory cytokine TGF-β, IL-10, and prostaglandin E from the infected macrophages [128]. However, further studies regarding the exogenous effect of LL-37 in severe pulmonary tuberculosis are warranted. Interestingly, administration of vitamin D3 or another potent inducer of LL-37, i.e., 4-phenyl butyrate (PBA), may be an alternative treatment method of tuberculosis [129].

## 5. Deregulations of HDPs Expression in Selected Diseases

### 5.1. Periodontal Diseases

An imbalanced unhealthy oral microbiota ushers the entry of various cariogenic, periodontal microbes which engenders oral biofilm formation and periodontal diseases such as gingivitis and periodontitis. The oral epithelial tissues, mainly the gingival epithelium, play a significant role in resisting the colonization of unfavorable oral pathogens. These tissues readily secrete beta-defensin peptides, as well as histatins, which are the major host defense proteins of the saliva that maintain homeostasis of oral microbiota [130]. A significant correlation is observed between elevated levels of hBD-2, hBD-4, and HNP4 in the oral mucosal epithelial cells of both adults and children with the development of dental caries. They are considered as important clinical biomarkers of periodontal diseases and dental caries. It has been shown that there is a declined expression of beta-defensin 1 mRNA gene in the inflamed gingival tissues and periodontal structures. Conversely, chronic cases of periodontitis manifest an elevated expression of the *hBD-1* gene [131,132]. The severity of periodontal diseases and dental plaques grows, along with a heightened expression of hBD-2 and histatin-5. Higher activity of pro-inflammatory cytokines modulates the progression of the infection, which further stimulates the production of the defensin peptides through various transcription factors [133]. Recently, 89 patients were monitored according to their periodontal status in relation to other clinical parameters [134]. This study identified an increase in the salivary production of hBD-2, triggered by inflammatory processes and pathogen derived metabolites that can be considered as a possible diagnostic biomarker s of periodontal diseases.

Healing of periodontal lesions is initiated by various growth factors, pro-inflammatory mediators, and antimicrobial peptides accumulating at the infected site. In detail, a complex network of highly specialized growth factors, namely, insulin-like growth factor (IGF1, IGF2), transforming growth factor (TGF-α, TGF-β), epidermal growth factor, and platelet-derived growth factor, coordinates the reparative process by rapid differentiation of keratinocytes and fibroblasts [135]. These growth factors also assist the wound healing mechanism by influencing the gene expression pattern of antimicrobial peptides that typically participate in the epithelial cell proliferation, migration, and inhibition of colonizing microbial pathogens at the site of injury. While it is established that wounding influences the expression of HDPs, not all of them function the same way. Recent reports have highlighted the distinct immune responses triggered within the wounded gingival epithelial cells (GECs) and gingival fibroblasts (HGFs) upon treatment with IGF1 and TGF-α. These growth factors enabled efficient wound closure and differently modulated the expression of hBD-2, CCL20, IL-1, and IL-8. The findings indicate that hBD-2 was exclusively enhanced in the gingival epithelial cells measured at set time points of 6 h and 24 h post-wounding, particularly in those cells associated with the keratinocyte differentiation marker involucrin. Additionally, hBD-2 along with CCL20, IL-1, and IL-8 control the invasion of bacterial microbes and impact the neutrophil defense mechanisms [136]. Contrarily, the wounded gingival fibroblasts (HGFs) witnessed a substantially low expression pattern of hBD-2 and CCL20, with or without growth factor treatment, that was suggested as a mechanism protecting fibroblast overgrowth into the epithelial wound [136]. fibroblast overgrowth into the epithelial wound.

### 5.2. Inflammatory Lung Diseases

Cystic fibrosis (CF) is a life-limiting disease characterized by recurrent respiratory infections and inflammation, connected to altered composition and volume of the airway surface liquid (ASL). For instance, a reduced bicarbonate HCO_3_^-^ secretion resulting in a decrease of airway surface pH (average 6.8–7.5) was observed. Interestingly, it was also found that the acidic pH weakened the action of LL-37 and hBD-3 against invasive *Staphylococcus aureus* and *Pseudomonas aeruginosa* infections by affecting their structural net charge. Therefore, it could be suggested that a similar mechanism of acidic pH-reduced antimicrobial activity may occur in other inflammatory conditions taking place in cerebral spinal fluid, peritoneal fluid, and pleural fluid. Additionally, it has been noted that high ionic strength (Na^+^, K^+^, Cl^+^) may impair the antimicrobial activity of hBD-2, lysozyme, and lactoferrin [137]. Moreover, CF patients suffer from viscous sputum that accumulates and obstructs their airways. The thick mucus is characterized by heterogenous complex aggregates of DNA and F-actin filaments derived from leukocytes that have encountered necrotic death. Thus, the antimicrobial function of LL-37, lysozyme, lactoferrin, and hBD-3 released in the respiratory airways is substantially hindered as they stabilize DNA/F-actin bundles. Additionally, neutralization of the immune function of neutrophil protease and IL-8 take place during DNA/F-actin bundles formation [138,139]. It is also worthwhile to underline that abundant secretion of cysteine cathepsins from the macrophages hinders the functional expression of hBD-2.

Chronic obstructive pulmonary disease (COPD), bronchitis, and asthma are all characterized by inflammation that develops as a consequence of pro-inflammatory mediator secretion. Immune cells distributed throughout the lungs are responsible for sudden exacerbations associated with the production of cytokines, oxidative stress, and protease secretion, including caspases, neutrophil elastase, and matrix metalloproteinases. One study reports the enhanced expression of hBD-2 in the distal airway epithelial cells of COPD patients, but a rather diminished expression of hBD-2 in the central airways, despite the exaggerated expression of TLR-4 receptors [140]. This distinct variation was found to be in correlation with exposure to cigarette smoking. While it is evident that every cell in the body requires ATP for its biological function of energy production and retention, little is known about its possible involvement in the immune system response to bacterial infection and inflammation. In a *P. aeruginosa*-infected rat model, ATP administration led to rapid stimulation of hBD-2 production. The mechanism of ATP action involved NADPH family of oxidases (DUOX 1) via ion channel receptors P2X, P2Y activation, and regulation of multiple signaling pathways ERK1/2 and NF-κB [141]. The released defensin peptide was found to control the inflammatory processes underlying the acute infection of pneumonia by suppression of TNF-α and IL-6. Furthermore, another study detected the potent ability of the IL-17 family of cytokines in the induction mechanism of the *hBD-2* gene. Typically, most of the immune cells, including T helper cells, macrophages, dendritic cells, and natural killer cells, secrete IL-17 family of cytokines. These cytokines act in concordance with the tumor necrosis factor and IL-1 to promote the induction of other inflammatory mediators production, which individually or collectively can stimulate the secretion of beta-defensins via the activation of various signaling pathways [142,143]. For example, the stimulatory functions of IL-17 in the airway epithelial cells promoting transcription of the *hBD-2* gene through the action of JAK and NF-κB signaling have been reported [144]. Moreover, IL-17 has an impact on other cytokines such as IL-1α, IL-β, IL-6, IL-7, and TNF-α, which contribute to the production of hBD-2. On the other hand, the alveolar macrophages and dendritic cells consistently maintain the release of IL-22. According to a recent study, in which alveolar epithelial cells (A549) were screened for the abundant display of IL-22 receptors and subjected to treatment with different doses of IL-22, an increase of *hBD-2* mRNA transcript synthesis via the STAT3 pathway was observed [145]. Thus, this study revealed a new immunomodulatory role of IL-22 in stimulation of the lung defensins in response to exposure to pathogenic bacteria and viruses. Interestingly, hBD-1 has also emerged as a clinical biomarker of COPD and other inflammatory lung diseases, such as asthma [146]. However, in this case, an altered expression of hBD-1 may be aggravated by gene copy number variations.

### 5.3. Inflammatory Bowel Diseases

The human defensins 5 and 6 (HD5, HD6) are particularly important in preserving the homeostatic equilibrium of the enteric mucosa layer, exhibiting different effects against the essential inducers of their secretion, i.e., various products of the Gram-positive and Gram-negative bacteria [147]. To illustrate, there is a remarkable reduction in the expression levels of HD5 and HD6 by the Paneth cells in inflammatory bowel conditions such as Crohn’s disease. This shift in expression could be attributed to the cause by genetic changes in the NOD2 receptor [148]. Furthermore, a recent study suggests the possibility of using other HDPs, such as the level of fecal HNP, as a non-invasive biomarker of intestinal inflammation in patients suffering from colitis ulcerosa [149].

The human beta-defensins are also naturally expressed in the epithelial cells of the gastric mucosa and extensively participate in host defense against *Helicobacter pylori* colonization, a bacterium present in a high proportion (~80%) of people throughout the world [150]. Multilevel signaling pathways promote the molecular mechanism of induction of beta-defensins in response to the initial stages of *H. pylori* infection. In addition, it has been shown that the phosphorylation of a serine residue of EGFR may modulate the release of hBD-3 [151], revealingan underlying interdependent relation between the stimulated transforming growth factor β-activated kinase-1 (TAK1), p38α pathway, and phosphorylation of EGFR receptor in the amplified release of hBD-3 in the gastric mucosa involved in *H. pylori* infection.

## 6. Conclusion

The multifunctional host defense peptides provide a link between innate and adaptive immunity against different microorganisms and contribute to inflammation of infected sites. Depending on the cell type and extracellular environment, some of these peptides exert contrasting functions, wherein they promote or suppress inflammatory processes. A strongly compromised action of host defense peptides against intruders and delayed resolution of inflammatory mediators underlies the development of inflammation in different diseases. Some of these peptides may serve as potential clinical biomarkers for a wide range of inflammatory diseases. Evidently, antimicrobial regulation is crucial to limit the exacerbation of inflammatory signaling molecules. While various factors govern the release of HDPs, any dysregulation can favor an imbalanced feedback mechanism between the host-induced anti-inflammatory and pro-inflammatory processes. In summary, a deeper understanding of the diverse functional roles of HDPs in the body’s physiological response to inflammation and disease is crucial and represents the first approach to develop new therapeutic strategies based on HDPs aimed at resolving the progression of inflammatory diseases and strengthening the host barrier defenses.

## Figures and Tables

**Table 1:** Host defense peptides—antimicrobial and immunomodulatory functions and disease association.

Class of Host Defense Peptides	Host Defense Peptides	Gene	Chromosome Location	Site of Expression	Biological Function	Dysregulated Expression of HDPs in Diseases	References
**Cathelicidins**	LL-37	*CAMP*	3p21.31	Innate immune cells Gut epithelial cells Respiratory system Salivary glands Skin	Wound healing and tissue repair LPS neutralization Recruitment of neutrophils Dendritic cells activation Intestinal barrier integrity Antiviral activity	Chronic intestinal infection↓ Systemic sclerosis↓ Chronic obstructive pulmonary disease↓ Psychological stress (murine CRAMP)↓ Atherosclerosis↑ Psoriasis↑	[18,19,23], [34,35,36,37]
**α-defensins**	HNP-1 HNP-2 HNP-3 HNP-4 HD-5 HD-6	*DEFA1* *DEFA3* *DEFA4* *DEFA5* *DEFA6*	8p23.1	Bone marrow Polymorphonuclear leukocytes Salivary glands Oronasal cavity and nasal mucosa Gastrointestinal and urinary tract Intestinal Paneth cells Bronchial cells Female reproductive system	Chemoattractant Phagocytosis induction Microbicidal activity Gut microbiota homeostasis Antifungal activity	Crohn’s disease↓ Graft-versus-host disease↓ Sepsis↑ Coronary heart disease↑ Systemic lupus erythematosus↑ Periodontal infections↓ Colorectal cancer↑	[16,17,21] [38,39,40]
**β-defensins**	hBD-1 hBD-2 hBD-3 hBD-4	*DEFB1* *DEFB2* *DEFB3* *DEFB4*	8p 23.1-p23.2 8p23.1-p22 8p23 8p23	Epithelial and blood cells Skin Gut epithelium Respiratory tract Bone marrow Epidermal keratinocytes Gingival epithelium Small intestine	Innate immune defense Wound healing Cytokine enhancement Dendritic cell modulation Neutrophil recruitment Pro-inflammatory mediator Antimicrobial activity	Oral squamous cell carcinoma↓ Liver cancer and colorectal cancer↓ Periodontitis↓ Asthma↑ Esophageal and cervical cancer↑ Interleukin-17A-mediated psoriasis↑ Ulcerative colitis↑ Chronic obstructive pulmonary disorder↑	[20,22], [41,42,43,44,45,46,47]
**Histatins**	His1His3His5	*HTN1* *HTN3* HTN3 *	4q13.3	Salivary glands	Oral health Wound healing	Aqueous deficient dry eye disease↓ Oral candidiasis↓	[48,49]
**RNases**	RNase 7	*RNASE7*	14q11.2	Skin Genito-urinary tract	Immunomodulatory	Allergic rhinitis↓ Urinary tract infections↑	[50,51]

Abbreviations: HNP-1, human neutrophil peptide 1; HNP-2, human neutrophil peptide 2; HNP-3, human neutrophil peptide 3; HNP-4, human neutrophil peptide 4; HD-5, human defensin 5; HD-6, human defensin 6; hBD-1, beta-defensin 1; hBD-2, beta-defensin 2; hBD-3, beta-defensin 3; hBD-4, beta-defensin 4; His1, histatin-1; His3, histatin-3; His5, histatin-5; RNase 7, ribonuclease 7; HTN3*, proteolytic variant of HTN3; ↑, upregulated; ↓, downregulated; HDPs, host defense peptides; LPS, lipopolysaccharides.

## Abbreviations

AMPs	Antimicrobial peptides
ATP	Adenosine triphosphate
BM	Bone morphogenetic protein
CAMP	Cathelicidin antimicrobial peptide
CCR2	C–C chemokine receptor type 2
CCR6	C–C chemokine receptor type 6
CNV	Copy number variation
COPD	Chronic obstructive pulmonary disease
DAMPs	Damage-associated molecular patterns
DC	Dendritic cell
DUOX1	Dual oxidase 1
EGFR	Epidermal growth factor receptor
ELA2	Neutrophil elastase 2
FGF2	Fibroblast growth factor
GEC	Gingival epithelial cell
HBEGF	Heparin binding EGF like growth factor
HER2	Human epidermal growth factor receptor 2
HGFs	Human gingival fibroblasts
HMGB1	High mobility group box 1
IDR	Innate defense regulator
IGF	Insulin like growth factor
IL-1α	Interleukin-1α
IL-33	Interleukin-33
JAK	Janus Kinase
LPS	Lipopolysaccharides
LTA	Lipoteichoic acid
MAPK	Mitogen-activated protein kinase
MIC	Minimum inhibitory concentration
MIP–3	Macrophage inflammatory protein-3 alpha
MRGPRX2	Mas-related G-protein coupled receptor member X2
NETs	Neutrophil extracellular traps
NF-κB	Nuclear factor kappa-light-chain-enhancer of activated B cells
NOD2	Nucleotide-binding oligomerization domain
OSCC	Oral squamous cell carcinoma
P2X7	Purinoceptor 7
SNP	Single nucleotide polymorphism
STAT	Signal transducer and activator of transcription
